# Length-weight relationship, condition factors and reproductive biology of the spineless cuttlefish *Sepiella inermis* (Ferussac & d’Orbigny, 1848) in the southeastern regions of the Bay of Bengal, Bangladesh

**DOI:** 10.1016/j.heliyon.2025.e42338

**Published:** 2025-02-04

**Authors:** Roksana Jahan, Md. Nasim Mahmud

**Affiliations:** a Department of Marine Fisheries and Oceanography, Sher-e-Bangla Agricultural University, Dhaka, Bangladesh; b Biological Oceanography Laboratory, Department of Marine Fisheries and Oceanography, Sher-e-Bangla Agricultural University, Dhaka, Bangladesh; c Department of Aquaculture, Sher-e-Bangla Agricultural University, Dhaka, Bangladesh

**Keywords:** *Sepiella inermis*, Length-weight relationship (LWRs), Negative allometry, Condition factors, Spawning season, Bay of bengal

## Abstract

*Sepiella inermis*, spineless and small-size cuttlefish, is one of the important species of cephalopods and widely distributed in the Indo-west Pacific region. This study, for the first time in the southeastern region of the Bay of Bengal, Bangladesh, was conducted to investigate the length-weight relationship, condition factors and spawning season of *S. inermis.* Samples (∼149) were collected monthly from the Bangladesh Fisheries Development Corporation (BFDC) landing center, Cox's Bazar from July 25, 2022 to May 15, 2023. Females were dominant throughout the year. Female exhibited higher dorsal mantle length (DML, 6.56 cm) and body weight (BW, 41.55 g) than males. *b* value of the length-weight (DML vs BW) relationship of female and male was 1.912 (r^2^ = 0.5072, *p* < 0.001) and 2.119 (r^2^ = 0.9557, *p* < 0.001), respectively, which indicated a negative allometric growth pattern. The Fulton's, relative condition factors and relative weight fluctuated based on sexes and seasons. Five gonadal (immature, maturing, mature, spawning, and spent) and four testis maturation stages were observed. The highest percentage of mature females (100 %) and males (75 %) were observed between 10-10.99 and 9–9.99 cm DML, respectively. 50 % of the females and males matured at 7.2 cm and 5.2 cm DML, respectively. On average, females have a greater gonadosomatic index (6.87 %) than males (1.98 %). A major peak in GSI of females was observed in September 2022, and second peak was in January 2023. The major spawning season in males was observed during January and May 2023. GSI had a negative correlation with condition factors in females and males. Since this is the baseline study on the reproduction aspects of *S. inermis*, further study could consider the effects of oceanographic and climatic parameters on the abundance and reproduction of cuttlefish in the Bay of Bengal.

## 1 Introduction

*Sepiella inermis,* usually known as spineless cuttlefish, is one of the important species of cephalopods. *Sepiella inermis* (Ferussac & d’Orbigny, 1848) is one of eight species in the genus *Sepiella* belonging to the family Sepiidae [1]. *S. inermis* is a small-sized cuttlefish (maximum mantle length about 12.5 cm) with narrow fins and a mantle with a pore at the posterior extremity [2]. This species presents a high tolerance for environmental parameters such as salinity and temperature [3] and is an important fishery resource in several areas of the globe [4]. The species is widely distributed through the Indo-West Pacific region from the Arabian Sea to Indonesian water and in the tropical waters of the southern South China Sea [2]. In Indian waters, it is widely distributed along both east and west coasts up to depths of about 40 m [5]. Though *S. inermis* were identified in Bangladeshi coastal waters, there is no detail study on reproduction and growth parameters of this species in Bangladesh.

There are several studies on length-weight relations (LWRs) on cuttlefish species [[6], [7], [8]] and *S. inermis* [[9], [10], [11]]. Onsoy and Salman [6] reported on 13,474 specimens belonging to 28 cephalopod species and found that most of cephalopod species showed negative allometry growth pattern (the exponent b is greater or lower than 3 considered to be positive or negative allometry, respectively; the value of b is exactly 3 represent isometry). In fact, using this terminology (isometry or allometry) it is difficult to identify the actual growth type of cephalopod species. Actually, evaluating LWRs in non-fish species poses challenges due to their unique body shape. Furthermore, unlike fish, cephalopods have unique life history traits such as semelparity, sexual dimorphism, rapid growth in relatively short life time, etc. [6]; all these factors might have large influence on the LWRs, and make this relationship in cephalopod more challenging. Noteworthy, the growth pattern is greatly influenced by reproductive stages of cephalopods. For instance, mature animals had comparatively lower b value than maturing, possibly due to the nature of semelparous cephalopod who stop or at least slow down growth when they reach maturity [6].

Besides length-weight relationships (LWRs), condition factors (i.e. Fultons K_F_ and relative condition factor K_r_) integrate key physiological components of organism's life history (e.g. growth rate and phenotypic variability) [12,13]. It offers a strong, accessible metric that managers can use to assess the overall health and fitness of organism populations as well as population-level response to ecosystem disturbance [14,15]. Condition factors have also indicated biochemical, ecological, and physiological processes in studies on the phenotypic responses of fish [16], crab [17], shrimp [18], and cephalopods [15,19] to environmental change. Several studies on condition factors of cuttlefish were conducted in the Yellow Sea, China [20], and the eastern Mediterranean [6]. The condition factors are generally influenced by the quantity and quality of organisms present, food availability, and the state of the aquatic environment [21]. These studies on cephalopods are quite challenging because (1) cephalopod do not have larval phase, unlike many fish species; this may affect the animals' condition unlike that of fish [6], (2) many of them stop feeding during the mating/spawning event, and (3) all coleoid cephalopods die after the reproduction ends (i.e. semelparity) [6].

Several research have focused on growth, abundance and reproduction on cuttlefish in the globe. For instance, growth studies were conducted in the Kakinada coast, India [9], Mumbai water [10], Thai waters [11]. The growth pattern of most of cephalopods vary temporally and spatially depending on temperature, salinity, depth, food availability, reproductive activity, size, sex, and season [5,22,23]. The reproductive biology of *Sepiella inermis* was carried out in several Indian water bodies including Mandapam [24], Thoothukkudi coast [25], Mumbai water [5], Ratnagiri, Arbian Sea, north-west coast of India [23]. The spawning period of *S. inermis* were also distinct to geographically. The spawning period lasted from October to January in the Mumbai water [5] and two peak spawning seasons reported in November and March in the Thoothukkudi coast, India [25]. Even though prolonged spawning period were also recorded in Mandapam, India [24]. To our best knowledge, there has been no study on reproduction biology of *S. inermis* in Bangladesh.

The purposes of the present study were to determine the length-weight relationship and various condition factors of *Sepiella inermis*, and to examine spawning season as well as its reproductive biology in the southeastern regions of the Bay of Bengal, Bangladesh. Temporal distribution of lengths, body weight, condition factors and gonadosomatic index (GSI) of *S. inermis* were also observed. The structure of the testis and ovaries for each maturity stage at different months and lengths was also examined which allows for a better understanding of reproduction in terms of maturity acquisition and reproduction cycle. Correlation among length-weight relationship, condition factors and reproduction of *S. inermis* were also discussed. The present research for the first time attempted to contribute to the knowledge of growth pattern and reproductive biology of *S. inermis* in the Bay of Bangladesh, Bangladesh.

## 2 Materials and methods

### 2.1 Identification of *Sepiella inermis*

**Fig. 1:**
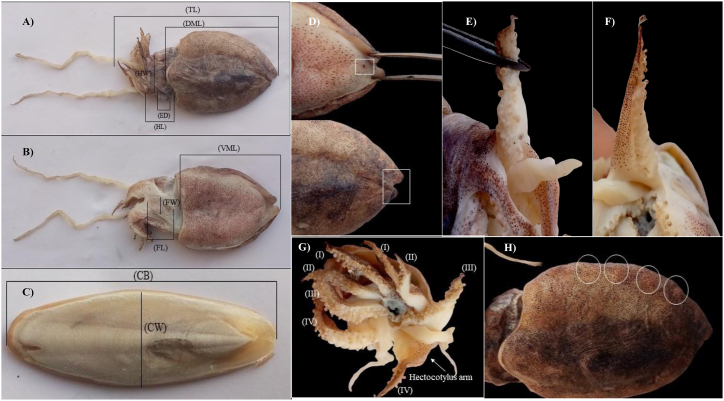
Identification of cuttlefish (*Sepiella inermis*). (A) measurement of different morphological characteristics such as total length (TL), dorsal mantle length (DML), head length (HD) and eye diameter (ED), (B) measurement of ventral mantle length (VML), funnel length (FL) and funnel width (FW), (C) measurement of cuttlebone (CB) and cuttlebone width (CW), (D) reddish pigmented gland pore on ventral mantle and tongue like projection on dorsal mantle, (E) arm sucker with tetra serial form, (F) hectocotylus on left ventral arm, (G) left arms (I,II,III,IV) and right arms (I,II,II,IV), (H) reddish patches adjacent to base of fins on dorsal mantle.

*Sepiella inermis* (Ferussac & d’Orbigny, 1848) was investigated and identified according to Jereb & Roper [2] (Fig. 1). It is a spineless cuttlefish with an oblong mantle (Fig. 1A and B). The outline of cuttlebone is broad and oval (Fig. 1C). A median rib is distinct in the dorsal part of cuttlebone. Granule present in the dorsal side of the cuttlebone. Spine absents. The shape of inner cone is U-shape posteriorly. The inner cone limbs are uniform width and narrow, and the outer cone is chitinous, spatulate and expanded. Posterior gland and gland pore are present (Fig. 1D). The color of gland pore is reddish. There is a hectocotylus on the left ventral arm (Fig. 1F). The color of this species is greyish brown. More than seven patches are found at the base of the dorsal mantle (Fig. 1H). Male and female specimens were distinguished by examining the ovary and testis after dissecting the cuttlefish.

### 2.2 Collection of samples of *S. inermis*

**Fig. 2:**
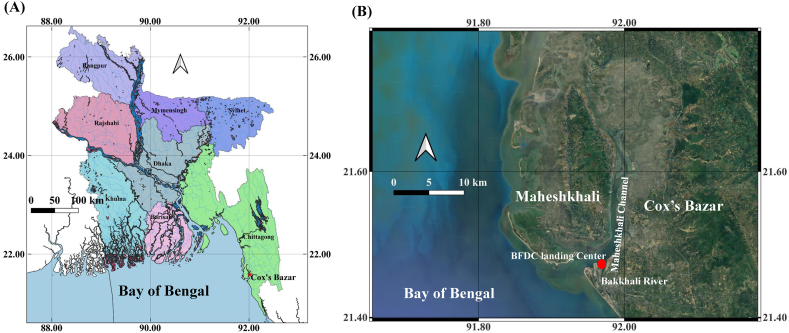
Map showing (A) the entire Bangladesh and Cox's Bazar (marked by red color), and (B) the BFDC landing center in the Maheshkhali Channel (marked by red color), Cox's Bazar, Bangladesh.

A total 149 number of samples (female 77, male 72) were collected from the BFDC (Bangladesh Fisheries Development Corporation) landing center, which is located on the southeast coast of the Bay of Bengal, Bangladesh from July 25, 2022 to May 15, 2023 (Fig. 2). Cephalopods are mainly caught by trawl nets, sometimes using marine set bag nets which are operating up to 20 m of depth in the Bay of Bengal (Bangladeshi coast) [26]. Artisanal fishing can be possible up to 40 m depth in the Bay of Bengal, Bangladesh. Note that, sample collection was not possible during June 2023 due to the marine banning period. In Bangladesh, banning period started yearly from 20 May to 23 July. For this, sample collection during July 2022 and May 2023 were conducted late and early in the month, respectively. Sampling in the remaining month was usually conducted from mid of the month. Measurement of morphometric characters and gonadal observations were completed within 10–15 days after sampling.

The cuttlefishes were transported using regular styrofoam box in an insulated chilled condition and the flake ice was used for maintaining the fish quality intake. Individuals were preserved frozen after capture and then measured in the laboratory for total length (TL, cm, from the base of the posterior part of the body to the tip of the arms), dorsal mantle length (DML, cm, from posterior end of the body to base of the head), body weight (BW, g), and gonad weight (GW, g). A standard scale and digital weight machine were used for estimating length and body weight, respectively. Electronic balance (Electronic precision balance, Model: FSH) was used to calculate weight of ovaries and testis weight.

### 2.3 Frequency distribution of length and body weight of *S. inermis*

The distribution of dorsal mantle length (DML) frequency of total, male and female of *S. inermis* were plotted using 1.0 cm class interval and the distribution of body weight frequency of total, male and female of *S. inermis* were plotted using 10.0 g class interval. The sex ratio (male: female) of *S. inermis* was measured monthly and the findings were evaluated using a Chi-square test (1:1, *p* < 0.05). The Chi-square test was conducted using the formula χ^2^ = ∑(O-E)/E, where χ^2^ is Chi-square, O is observed value and E is expected value.

### 2.4 Length-weight relationship, condition factors and prey-predator's status of *S. inermis*

Equation W = *a* × L^*b*^ was used to examine the relationship between dorsal mantle length and body weight, where W is the body weight (BW, g), L is the dorsal mental length (DML, cm), and the regression parameters are *a* and *b*. Linear regression analysis is used to drive the a and b of the length-weight relationship: ln (W) = ln (*a*) + *b* ln (L) [27]. The Fulton's condition factor (K_F_) was computed using Fulton's formula: K_F_ = 100 (W/L^3^) (where W is the BW and L is DML). Furthermore, the relative condition factor (K_r_) was calculated using Le Cren's [14] formula: K_r_ = W/(*a* × L^*b*^), where W is the BW, L is the DML, and *a* and *b* are the LWR parameters. Relative weight (W_R_) was calculated as W_R_ = (W/W_s_) × 100 [27], where W is the body weight of a single species and W_s_ is the predicted normal weight as defined by W_s_ = *a* × L^*b*^ (here, the values of *a* and *b* are determined from the DML vs BW equation).

### 2.5 Observation of gonad and determination of gonadosomatic index (GSI) of *S. inermis*

The reproductive organs of the specimens were examined macroscopically to determine their sex. The weight of ovary and testis were measured to the closest 0.1 g. The maturity for each sex were assessed according to Gabr et al. [28]. Males and females maturity sacks are classified as immature (stage I), maturing (stage II), mature (stage III), spawning (stage IV), and spent (stage V). The spawning season was determined according to the gonadosomatic index (GSI) values. GSI was measured as the percentage of the ovary/testis weight in relation to the body weight (BW). GSI was calculated as Gonadosomatic index (GSI) = (Weight of gonad/Body weight) × 100.

### 2.6 Data analysis

Boxplot graphs were conducted using “ggplot2” packages in R, version 3.5.2 (R development core Team 2019). Prior to analysis, the normality test (Shapiro-wilk) of the data was conducted, and revealed that the data-set of this study was not normally distributed. Spearman correlation was used to determine the relationship among dorsal mantle length (DML), body weight (BW), Fulton's condition factor (K_F_), relative condition factor (K_r_) and relative weight (W_R_) using the “PerformanceAnalytics” packages in R. Kruskal-Wallis test was performed to determine the significance difference (*p* < 0.05) among the gonadosomatic index (GSI) values of different months. This test was conducted in IBM-SPSS Statistics 21. The principal component analysis (PCA) was performed using the “GGally”, “factoextra” and “ggfortify” packages in R.

### 2.7 Ethical statement

The Ethics Committee of the Department of Marine Fisheries and Oceanography, Sher-e-Bangla Agricultural University (SAU), Bangladesh, approved the experimental conception and execution after it satisfied the committee's standards. The cuttlefish sampling protocol strictly followed by the ARRIVE 2.0 guidelines [29]. The study adhered to the guidelines outlined in the “Guide for the care and use of Laboratory Animals” provided by the National Institutes of Health. The study did not involve the use of endangered or protected species.

## 3 Results

### 3.1 Monthly variation and frequency distribution of total length, dorsal mantle length and body weight of cuttlefish (*Sepiella inermis*) in the southeastern region of the Bay of Bengal, Bangladesh

**Fig. 3:**
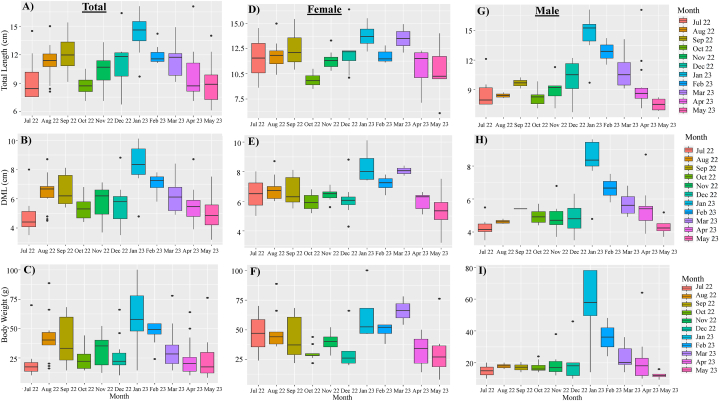
Monthly variation of Total length (TL, cm), Dorsal mantle length (DML, cm) and Body weight (BW, g) of total (A, B and C), female (D, E and F) and male (G, H and I) cuttlefish (*Sepiella inermis*) during July 25, 2022 to May 15, 2023 in the southeastern region of the Bay of Bengal, Bangladesh.

**Fig. 4:**
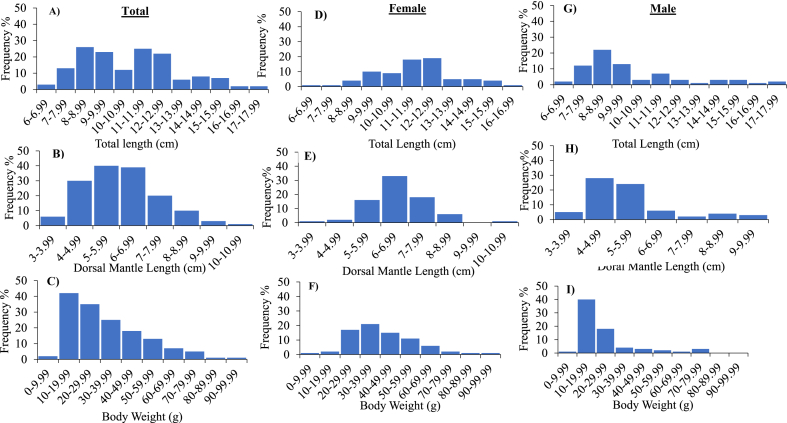
Frequency distribution of Total length (TL, cm), Dorsal mantle length (DML, cm) and Body weight (BW, g) of total (A, B and C), female (D, E and F) and male (G, H and I) cuttlefish (*Sepiella inermis*) during July 25, 2022 to May 15, 2023 in the southeastern region of the Bay of Bengal, Bangladesh.

For total cuttlefish, the average total length (TL) was 10.73 cm, with minimum and maximum lengths being 6.10 cm in May 2023 and 17.10 cm in April 2023, respectively (Fig. 3A). The range of 8–8.99 cm exhibited the largest frequency of TL (17.4 %), followed by 11–11.99 cm (16.7 %), 9–9.99 cm (15.4 %) and 12–12.99 cm (14.7 %) (Fig. 4A). The average value of dorsal mantle length (DML) measured 6.004 cm, with the lowest and greatest DML being 3.20 cm in May 2023 and 10.10 cm in January 2023, respectively (Fig. 3B). The highest frequency of DML (26.8 %) was found in the range of 5–5.99 cm, followed by 6–6.99 cm (26 %), 4–4.99 cm (20 %), and 7–7.99 cm (13.4 %) (Fig. 4B). The body weight (BW) that was measured ranged from 8.0 g in May 2023 to 99.9 g in January 2023, with an average of 32.9 g (Fig. 3C). The highest (28 %) and lowest frequency (0.7 %) of BW were observed with the range of 10–19.99 g and 90–99.9 g, respectively (Fig. 4C).

For females, the mean TL was 11.56 cm, and the lowest and highest lengths were 6.10 cm in May 2023 and 16.40 cm in December 2022, respectively (Fig. 3D). The range of 12–12.9 cm revealed the highest frequency of TL (24.6 %), followed by 11–11.99 cm (23.3 %) and 10–10.9 cm (11.6 %) (Fig. 4D). The DML measured 6.56 cm on average, with the smallest value being 3.20 cm in May 2023 and the greatest value being 10.10 cm in January 2023 (Fig. 3E). The highest range of DML was observed at 6–6.99 cm (42.8 %), followed by 7–7.99 cm (23.3 %) and 5–5.99 cm (20.7 %) (Fig. 4E). The average BW was 41.55 g, with minimum and maximum of 8.0g in May 2023 and 99.90 g in January 2023, respectively (Fig. 3F). The highest frequency (27.2 %) of BW was observed at a range of 30–39.99 g, followed by 20–20.99 g (22 %), and 40–40.99 g (19.4 %) (Fig. 4F).

For males, the mean TL was 9.83 cm, with a minimum length of 6.70 cm in December 2022 and a maximum length of 17.10 cm in April 2023 (Fig. 3G). The highest frequency of TL (30.5 %) was observed at a range of 8–8.9 cm, followed by 9–9.99 cm (18 %), 11–11.99 cm (9.7 %) and 10–10.99 cm (4 %) (Fig. 4G). The DML obtained an average value of 5.40 cm, with minimum and highest values of 3.50 cm in December 2022 and 9.60 cm in January 2023, respectively (Fig. 3H). The range of 4–4.99 cm had the largest frequency of DML (38.8 %), while 7–7.99 cm (2.7 %) exhibited the lowest frequency (Fig. 4H). The average value of BW was 23.64 g, with a minimum of 9.50 g in May 2023 and a maximum of 78 g in January 2023 (Fig. 3I). The highest frequency (55.5 %) of BW was observed at a range of 10–19.99 g, followed by 20–20.99 g (25 %), 30–39.99 g (5.5 %) (Fig. 4I). It was observed that females were dominant throughout the year. The sex ratio (male: female) ranged between 1:0.28 (March 2023) to 1:6 (September 2022). The sexes were significantly different (*p* < 0.05) in August 2022, September 2022 and April 2023 (Table 1).

**Table 1:** The sex ratio of cuttlefish (*Sepiella inermis*) during July 25, 2022 to May 15, 2023 in the southeastern region, Bay of Bengal, Bangladesh.

Months	Total	No. of male	No. of female	Sex ratio (Male/female)	Chi- square (df = 1)	*P* value	Significance
July 2022	8	6	2	1:0.33	2.00	0.1573	NS
August 2022	16	3	13	1:4.33	6.25	0.0124	S
September 2022	14	2	12	1:6	7.143	0.0075	S
October 2022	21	12	9	1:0.75	0.429	0.5127	NS
November 2022	14	6	8	1:1.33	0.286	0.5930	NS
December 2022	15	7	8	1:1.14	0.067	0.7963	NS
January 2023	12	8	4	1:0.5	1.333	0.2482	NS
February 2023	10	2	8	1:4	3.60	0.0578	NS
March 2023	9	7	2	1:0.28	2.778	0.0956	NS
April 2023	20	15	5	1:0.33	5.00	0.0253	S
May 2023	10	4	6	1:1.5	0.40	0.5271	NS
Total	149	72	77	1:1.06	0.168	0.6821	NS

Note: NS and S denote as non-significance and significance, respectively.

### 3.2 Length-weight relationship of cuttlefish (*Sepiella inermis*) *in the southeastern region, Bay of Bengal, Bangladesh*

**Fig. 5:**
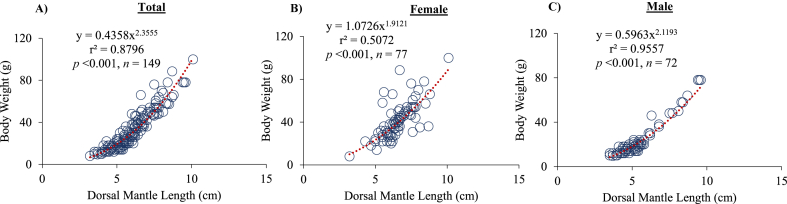
Length-weight relationships (DML vs BW) of total (A), female (B) and male (C) cuttlefish (*Sepiella inermis*) during July 25, 2022 to May 15, 2023 in the southeastern region, Bay of Bengal, Bangladesh.

There was a strong positive relationship between dorsal mantle length and body weight (r^2^ = 0.87, *p* < 0.001; r^2^ = 0.50, *p* < 0.001; r^2^ = 0.95, *p* < 0.001 for total, female and male, respectively) (Fig. 5A, B, C). The representation of negative allometry by the value of *b* was consistently 2.35, 1.91, and 2.11 for total, female, and male of *S. inermis*, respectively. The values of *a* were 0.44, 1.10, and 0.59 for total, female and male of *S. inermis*, respectively.

### 3.3 Condition factors and relative weight of cuttlefish (*Sepiella inermis*) in the southeastern region, Bay of Bengal, Bangladesh

**Fig. 6:**
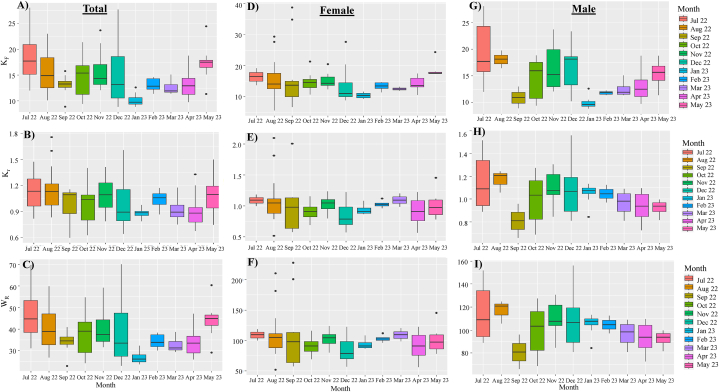
Fulton's condition factor (K_F_), relative condition factor (K_r_) and relative weight (W_R_) of total (A, B and C) female (D, E and F) and male (G, H and I) cuttlefish (*Sepiella inermis*) during July 25, 2022 to May 15, 2023 in the southeastern region, Bay of Bengal, Bangladesh.

For total, the K_F_, K_r_, and W_R_ had average values of 14.4, 1.00, and 37.15, respectively (Fig. 6A, B, C). The highest value of K_F_ (27.98) was found in July 2022, while the maximum values of K_r_ (1.75) and W_R_ (69.88) were found in August and December 2022 respectively. The lowest K_F_ value was 8.81 in January 2023, whereas the lowest K_r_ and W_R_ values were 0.59 and 22.83 in September 2022. The average values for K_F_, K_r_, and W_R_ in females were 14.93, 1.00, and 100.73, respectively (Fig. 6D, E, F). The highest values for K_F_, K_r_, and W_R_ (38.72, 2.27, and 227.47) were detected in September 2022. In August 2022, the lowest values of K_F_, K_r_, and W_R_ (5.46, 0.51, and 51.87) were observed. The average values for K_F_, K_r_, and W_R_ in males were 14.36, 1.01, and 101.39 respectively (Fig. 6G, H, I). The highest value of K_F_ (27.98) was found in July 2022, while the maximum values of W_R_ (155.80) and K_r_ (1.55) were found in December 2022. In January 2023, the lowest K_F_ (8.8) value, while September 2022 saw the highest values of K_r_ and W_R_ (0.65 and 65.75).

### 3.4 Maturity stages of female and male cuttlefish (*Sepiella inermis*) in the southeastern region, Bay of Bengal, Bangladesh

**Fig. 7:**
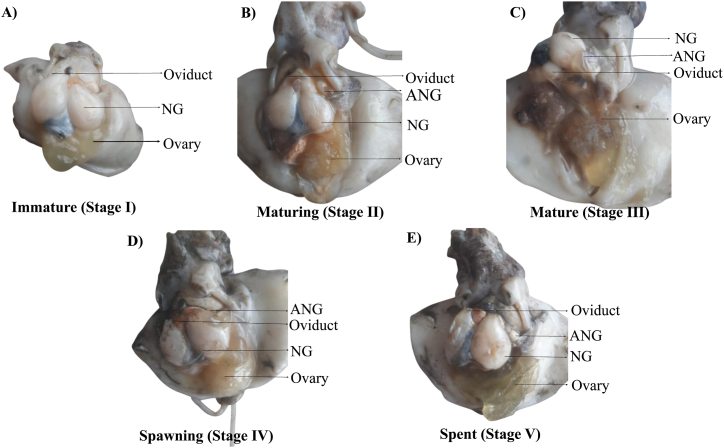
Macroscopic maturity stages of female cuttlefish (*Sepiella inermis*) during July 25, 2022 to May 15, 2023 in the southeastern region, Bay of Bengal, Bangladesh. A) Immature (Stage I), B) Maturing (Stage II), C) Mature (Stage III), D) Spawning (Stage IV), and E) Spent (Stage V). Note: NG and ANG represent Nidamental glands and Accessory Nidamental glands, respectively.

**Fig. 8:**
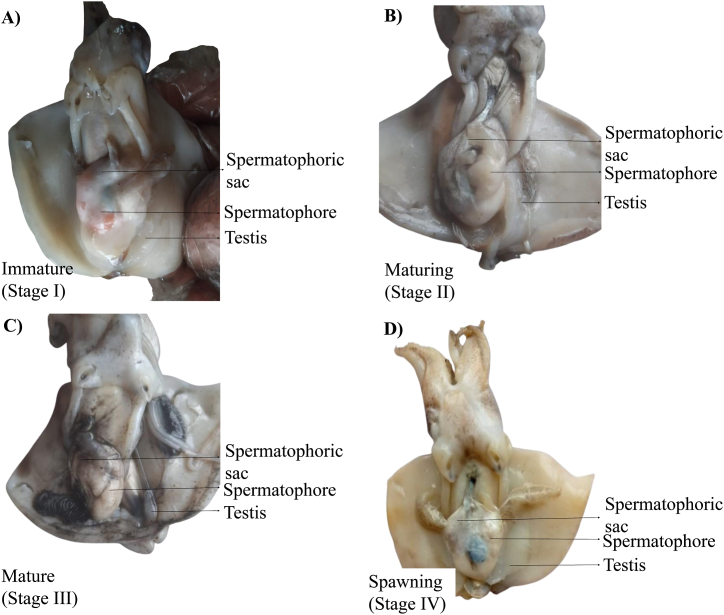
Macroscopic maturity stages of male cuttlefish (*Sepiella inermis*) during July 25, 2022 to May 15, 2023 in the southeastern region, Bay of Bengal, Bangladesh. A) Immature (Stage I), B) Maturing (Stage II), C) Mature (Stage III), and D) Spawning (Stage IV).

The macroscopic images of different maturity stages of female and male *Sepiella inermis*, were shown in Fig. 7, Fig. 8. In immature (stage I), the ovary was very small and disk-shaped. The nidamental glands are small in size and there doesn't seem to be any noticeable accessory nidamental gland (Fig. 7A) and the testis appeared slender and flat (Fig. 8A). In maturing (stage II), the ovary contained uniformly sized maturing yellowish eggs but this stage is short-lived. The nidamental glands were larger and thicker and had a creamy white color (Fig. 7B), while the testis became comparatively larger and thicker with a visible spermatophoric sac (Fig. 8B). The ovary was highly noticeable in mature (stage III), filled with numerous translucent eggs. The ovary had large, thick, creamy white nidamental glands, accessory nidamental glands with orange hue, and mature, clear, fully developed eggs (Fig. 7C). The testis was fully mature and creamy white, the spermatophores neatly packed within a spermatophoric sac (Fig. 8C). In spawning (stage IV) the ovary holds a limited number of loosely arranged striped eggs and a few mediums to small eggs that are connected to the ovarian tissue (Fig. 7D), while in male the spermatophoric sac swollen and full of spermatozoa (Fig. 8D). In spent (stage V), the ovary was composed of gelatinous material, devoid of eggs, flaccid nidamental glands and had a yellowish hue (Fig. 7E).

### 3.5 Monthly variations of relative proportion of each gonadal maturation stage of cuttlefish (*Sepiella inermis*) in the southeastern region, Bay of Bengal, Bangladesh

**Fig. 9:**
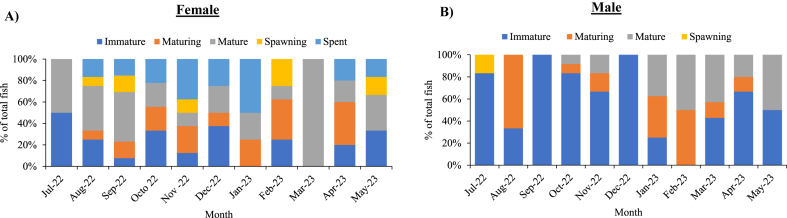
Monthly variations of relative proportion (%) of each gonadal maturation stage (for ovaries and testis) of female (A) and male (B) cuttlefish (*Sepiella inermis*) during July 25, 2022 to May 15, 2023 in the southeastern region, Bay of Bengal, Bangladesh.

The immature females *Sepiella inermis* (stage I) was observed in eight months except January 2023, March 2023 and April 2023 (Fig. 9A). The highest percentage of immature females (50 %) was found in July 2022, followed by December 2022 (37.5 %) and October 2022 (33.33 %). The female organisms showed the highest percentage (40 %) of maturing stage (II) in April 2023, followed by February 2023 (37.5 %), and January 2023 (25 %). Mature *S. inermis* (stage III) was found throughout the year, with the highest percentage of 100 % in March 2023. The highest proportion (25 %) of spawning *S. inermis* (stages IV) recorded in February 2023, followed by May 2022 (16.66 %) and September 2022 (15.38 %). The highest (50 %) and lowest percentage (15.38 %) of spent was observed in January 2023 and September 2022, respectively (Fig. 9A).

In males, the immature *Sepiella inermis* (stage I) was found throughout the year (Fig. 9B). In September 2022 and December 2022, the highest value was 100 %, while in August 2022, the lowest value was 25 %. The greatest (66.66 %) and lowest value (8.33 %) of maturing *S. inermis* (stage II) was observed in August 2022 and October 2022, respectively. Mature *S. inermis* (stage III) was found in October 2022, November 2022, January 2023, March 2023, and April 2023. The highest (50 %) and lowest percentages (8.33 %) of mature males were observed in May 2023 and October 2023, respectively. The spawning stage (IV) was found in July 2022. There was no spent stage (V) found possibly due to insufficient sample size (Fig. 9B).

### 3.6 Relationship between dorsal mantle length and relative proportion of each gonadal maturation stages of cuttlefish (*Sepiella inermis*) in the southeastern region, Bay of Bengal, Bangladesh

**Fig. 10:**
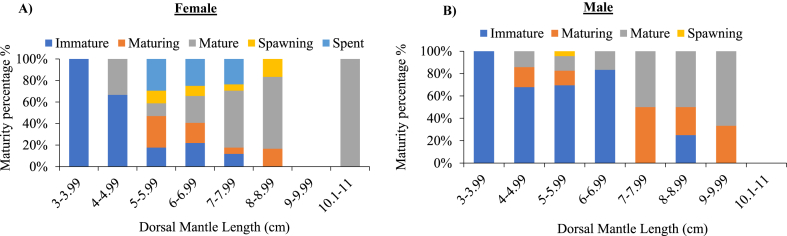
Relationship between dorsal mantle length (DML, cm) and relative proportion (%) of each gonadal maturation stages (for ovaries and testis) of female (A) and male (B) cuttlefish (*Sepiella inermis*) during July 25, 2022 to May 15, 2023 in the southeastern region, Bay of Bengal, Bangladesh.

**Fig. 11:**
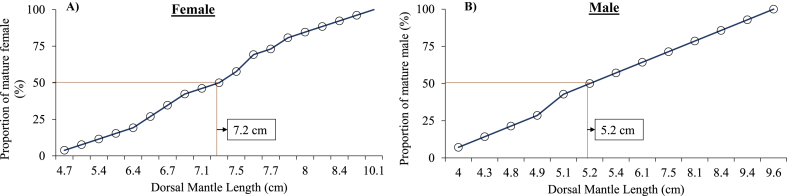
Size at first maturity of females (A) and males (B) of cuttlefish (*Sepiella inermis*) during July 25, 2022 to May 15, 2023 in the southeastern region, Bay of Bengal, Bangladesh.

The immature females were observed between 3 and 8 cm in DML (Fig. 10A). The majority of immature female (100 %) were recorded at 3–4 cm DML, followed by 4–5 cm (66.66 %) and 6–9 cm (21.87 %) DML. The maturing females were observed from 5 to 9 cm in DML, with the highest and lowest proportions recorded at 5–6 cm (29.41 %) and 6–7 cm (18.75 %) in DML, respectively. Mature females were found from 4 to 11 cm in DML, with the highest and lowest percentage recorded being 10–11 cm (100 %) and 5–6 cm (11.76 %) in DML, respectively. Spawning stages of females were observed between 5 and 9 cm in DML, with the highest and lowest proportion recorded at 8–9 cm (16.66 %) and 7–8 cm (5.88 %) in DML, respectively. Spent stages of females were found between 5 and 8 cm in DML. Immature males were observed ranged from 3 to 9 cm in DML, with the highest and lowest proportions observed at 3–4 cm (100 %) and 8–9 cm (25 %) in DML (Fig. 10B). Maturing males were observed between 4 and 9 cm in DML, with the highest and lowest proportion shown at 7–8 cm (50 %) and 5–6 cm (13.04 %) in DML. Mature males were observed between 4 and 10 cm in DML, with the maximum and lowest proportions at 9–10 cm (75 %) and 4–5 cm (14.28 %) in DML, respectively. At 5–6 cm in DML, the spawning stage of males were observed. The maturity curve showed that 50 % of the females matured at 7.2 cm and males matured at 5.2 cm (Fig. 11A and B).

### 3.7 Monthly variations of gonadosomatic index of cuttlefish (*Sepiella inermis*) in the southeastern region, Bay of Bengal, Bangladesh

**Fig. 12:**
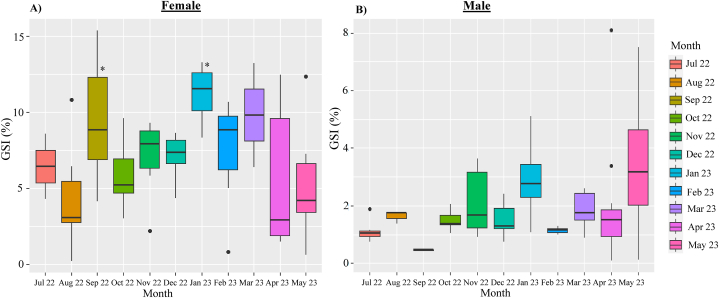
Monthly variations of gonadosomatic index (GSI, %) of female (A) and male (B) cuttlefish (*Sepiella inermis*) during July 25, 2022 to May 15, 2023 in the southeastern region, Bay of Bengal, Bangladesh. Asterisk sign represent statistical difference at *p* < 0.05.

Females had a greater average GSI (6.87 %) than males (1.98 %) (Fig. 12A). The first GSI peak in females was found in September 2022 (9.13 %). The GSI falls from October 2022 to December 2022, from 6.35 to 2.38 %. The second highest peak (9.78 %) was recorded in January 2023, followed by February 2023 (9.02 %) and March 2023 (7.05 %). The GSI again fallen from March 2023 (7.05 %) to May 2023 (5.33 %). The GSI values in September 2022 and January 2023 showed significant differences compared to other months (*p* < 0.05) (Fig. 12A). GSI in male increased from October 2022 (1.49 %) to January 2023 (2.90 %), with January 2023 exhibiting the first peak. Between March 2023 and May 2023, the GSI increased once more and second peak was found in May 2023 (3.49 %) (Fig. 12B). The GSI values for males showed no significant differences among months (*p* < 0.05) (Fig. 12B).

### 3.8 Relationship among dorsal mantle length, body weight, condition factors and gonadosomatic index of cuttlefish (*Sepiella inermis*) in the southeastern region, Bay of Bengal, Bangladesh

**Fig. 13:**
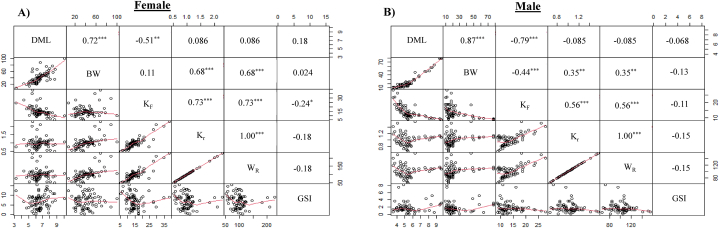
Spearman correlation among dorsal mantle length (DML), body weight (BW), Fulton's condition factor (K_F_), relative condition factor (K_r_), relative weight (W_R_) and gonadosomatic index (GSI) of female (A) and male (B) cuttlefish (*Sepiella inermis*) during July 25, 2022 to May 15, 2023 in the southeastern region, Bay of Bengal, Bangladesh. Note: ∗, ∗∗ and ∗∗∗ denote *p* < 0.05, <0.01 and < 0.001, respectively.

In females, there was a significant positive correlation between BW and DML (r = 0.72, *p* < 0.001) (Fig. 13A). K_F_ was significant negative and positive correlation with DML (r = −0.51, *p* < 0.01) and BW (r = 0.11, *p* = 0.331), respectively. W_R_ and K_r_ significantly significantly positively with BW and K_F_ (r = 0.68, *p* < 0.001 and r = 0.73, *p* < 0.001 respectively). The results show that GSI had a positive correlation with DML (r = 0.18, *p* = 0.127) but a negative correlation with K_F_, K_r_, and W_R_ (r = −0.24, *p* < 0.05; r = −0.18, *p* = 0.125 and r = −0.18, *p* = 0.125, respectively). In male, BW has a strong positive correlation with DML (r = 0.87, *p* < 0.001), but K_F_ had a significant negative correlation with both DML and BW (r = −0.79, *p* < 0.001 and r = −0.44, *p* < 0.001, respectively (Fig. 13B). K_r_ and W_R_ significantly positively related to BW, and K_F_ (r = 0.35, *p* < 0.01 and r = 0.56, *p* < 0.01, respectively). GSI positively correlated but negatively correlated with K_F_, K_R_, and W_R_ (r = −0.11, *p* = 0.337; r = −0.15, *p* = 0.224 and r = −0.15, *p* = 0.224, respectively).

**Fig. 14:**
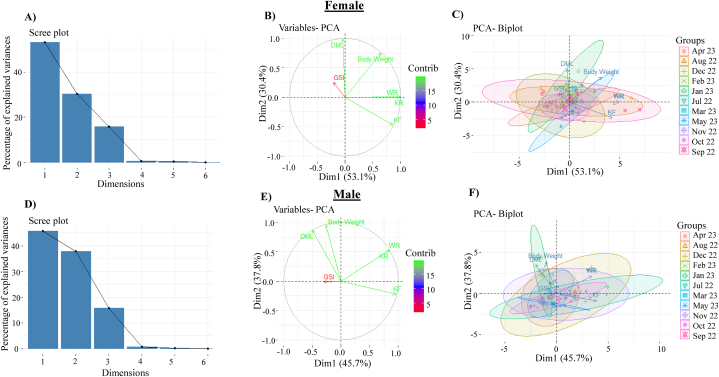
Principal component analysis (PCA) of dorsal mantle length (DML), body weight (BW), Fulton's condition factor (K_F_), relative condition factor (K_r_), relative weight (W_R_) and gonadosomatic index (GSI) of female and male cuttlefish (*Sepiella inermis*) during July 25, 2022 to May 15, 2023 in the southeastern regions of the Bay of Bengal, Bangladesh. Scree plot of principal component analysis (PCA) of female (A) and male (D) cuttlefish (*Sepiella inermis)*. Variables of first principal component and second principal component in PCA of female (B) and male (E) cuttlefish (*Sepiella inermis*). Biplots of first principal component and second principal component in PCA of female (C) and male (F) cuttlefish (*Sepiella inermis*).

In female, first two axes of PCA explain 83.5 % of total variance (Fig. 14A and B). PCA 1 accounted for 53.1 % of variance, while PCA 2 accounted for 30.4 % of the variance. BW, K_F_, K_r_ and W_R_ showed significant positive relationship, and DML and GSI exhibited negative relationship with PCA 1. Biplot of PCA 1 and PCA 2 showed that different months exhibited overlapping throughout the study period (Fig. 14C). The first two PCA axes in males explain 83.5 % of all variances (Fig. 14D and E). PCA 1 and PCA 2 explained 45.7 % and 37.8 % of the variance, respectively. PCA 1 revealed significant positive relationship with K_F_, K_r_ and W_R_, and negative relationship with BW, DML, and GSI. Throughout the study period, distinct months showed overlapping in the biplot of PCA 1 and PCA 2 (Fig. 14F).

## 4 Discussion

The present study was the first attempt in the southeastern region of the Bay of Bengal, Bangladesh that provided valuable information about the L-W relationship, condition factors, and reproductive biology of *Sepiella inermis*. In L-W relationship, males and females showed negative allometric growth pattern of *S. inermis*. The Fulton's and relative condition factor, and relative weight fluctuated based on sexes and months. According to the gonadosomatic indices (GSI), two major peaks of spawning season in female was observed in September 2022 and January 2023, while male exhibited two spawning seasons during January and May 2023. This is the baseline study on the reproductive aspects of *S. inermis*.

Consistent with previous records (*b* = 1.932 and 2.321 for males and females in Mandapam and Rameswaram, India [24]; *b* = 2.2080 and 2.3016 form males and females in Kakinada coast, India [9]), LWRs of *S. inermis* showed negative allometric (*b* = 2.120 and 1.912) for males and females, respectively. Two possibly caused for this growth pattern such as (1) types of sexes and maturation stages, and (2) its unique physiological characteristics. Firstly, it is revealed that coefficient *b* values of male was higher than female, and consistently, female exhibited heavier than male, possibly due to presence of gonad in female. The present study exhibited that weight of gonad may cover 2.02 %–13.24 % of females body weight when reaching maturity, whereas males constitute 1 %–3.7 % of body weight. In fact, the reproductive organs of females become heavier at maturity that influencing the overall weight which slows down the growth rate of females more rapidly than that of males [30,31]. Coefficient *b* values of mature female animals were found lower than that of maturing female animals [6]. This might be caused by feeding stops in reproduction season [6,32,33]. After stop feeding, the energy needs of animals are compensated by consuming the energy reservoirs i.e. fat, muscles, digestive glands, etc. [6]. Therefore, it is obvious caused dramatically loss of weight and thus affect the animals' body condition as well as LWRs of cuttlefish. Secondly, unique growth type and body structure of cephalopods than fish. Cephalopods have different body parts that different growth rates i.e. arms, head and mantle that influence the weight values so make the LWRs difficulty to apply term 'isometry' and 'allometry" [6,34]. The cephalopod species has an early rapid growth phase (exponential) and then a decreasing growth rate (logarithmic) gradually after reaching maturing [6]. Further research are needed to evaluate this phenomena of cephalopod species in the Bay of Bengal, Bangladesh.

The range of Fulton's condition factor (14.4, from minimum 8.81 to maximum 27.98) is consistent with previous findings in the eastern Mediterranean [6]. For instance, Onsoy and Salman [6] observed Futon's condition factors that varies from 3.02 to 31.31 (mean 12.51 ± 02.61) in Sepiida and from 9.16 to 125.55 (mean 31.03 ± 12.48) in Sepiolida. Consistent with previous studies [35] the mean K_r_ value was 1.00 (0.59–1.75), suggested a state of well-being for the species. The deviation of K_r_ from 1 reveals information concerning the difference in food species [14]. Since the coastal and offshore waters of the Bay of Bengal are rich in crustaceans, bony fish, mollusk. polycreates and worms [36,37], these organisms support the growth of *S. inermis*. Condition factors also varied at different points of the development cycles. Unlike fish (K_F_ values positively related with spawning [38], K_F_ and K_r_ of *S. inermis* negatively correlated with spawning (GSI) (Fig. 10a, b), possibly due to feeding stops during spawning stages, as explained before. Relative weight (W_R_) helps estimate the status of prey predators and ecosystem disturbances at the population level [39]. Low W_R_ values (<100) for individuals or populations indicate problems like low prey accessibility or high predator density. In contrast, high value (>100) indicates a surplus of prey or low predator density [39]. The average value of W_R_ of *S. inermis* were lower than 100 in this study, suggesting cuttlefish would be suitable prey for marine predators in the Bay of Bengal.

In this study, the sex ratio of *S. inermis* showed dominance of female throughout the year. The variation in the sex ratio may be due to movement of the females to inshore waters for spawning [5]. Mature stages of female and male were observed at the wide range of DML that could suggest semelparity in the species [25]. The size at first maturity in the present study was estimated at 52 mm for males and 72 mm for females, but according to Unnithan [24], Sarvesan [40] and Neethisalvan et al. [25], the values are 51 mm, 47 mm and 45 mm for males and 31 mm, 58 mm, 45 mm for females, respectively. From the observations made, it seems that the species in northeastern coast of Bangladesh attains maturity at a comparatively larger size than other places (i.e. east coast in India [5]). This statement proposed further studied to include more samples and broad range of sampling sites in the Bay of Bengal.

As the occurrence of mature specimen and higher values of GSI in male and female *S. inermis* were observed throughout the study period indicated prolonged spawning of *S. inermis* in the Bay of Bengal, with two peaks in September and January for females, and in January and May for males. This finding is consistent with Thoothukkidu in south east coast of India, where prolong breaded were observed with two peak spawning period, one in November and another in March [25]. It is attributed that proportion of mature female were comparatively higher than male. Overall, suitable physic-chemical parameters and food availability are main factors for growth and breeding of *S. inermis*. Consistent with marine fishes (i.e. Hilsha) in the Bay of Bengal, Bangladesh, two peaks spawning periods are obvious generally in monsoon and post-monsoon [41]. Typically, the monsoon in Bangladesh characterized by higher river discharge, nutrients, and food availability such as fish, crustaceans, algae, etc. [42,43]; all these factors suitable for growth and breeding of marine organisms. Moreover, average water temperature in the Bay of Bengal ranged from 26 °C in winter to 29.6 °C in summer [44], which supports the breeding temperature of *S. inermis*. Because the average breeding temperature of *S. inermis* is 28 °C [45]. Noteworthy, rainfall, lunar periodicity, length of day, water current, freshwater flow, turbidity, sandy bottom, and circular current might also influence the breeding and migration of marine species in the Bay of Bengal, Bangladesh [46]. This study is limited to avoiding oceanographic and meteorological parameters that could affect the abundance and reproductive biology of *S. inermis* in the Bay of Bengal. However, the percentage of spent females was relatively less compared to other gonadal stages during the study period, attributed to post-spawning exhaustion of cuttlefish and the semelparous life cycle that individuals die after spawning [25,47].

## 5 Conclusion

This is the first attempt to study the reproduction aspects of *S. inermis* in the southeast coastal waters of the Bay of Bengal, Bangladesh. The negative allometry growth pattern was observed in males and female cuttlefish. Based on gonadosomatic indices (GSI), two major peaks of spawning season in female were observed in September 2022 and January 2023, while male exhibited two spawning seasons during January and May 2023. The results presented here contribute to the basic knowledge needed for sustainable fisheries and biodiversity conservation goals. Since this study was restricted to limited samples and lacked environmental variables, further study could suggest that the effects of various physical-chemical oceanographic parameters and biological interaction on the abundance and reproduction of cuttlefish as well as other cephalopod species in the Bay of Bengal.

## CRediT authorship contribution statement

**Roksana Jahan:** Writing – review & editing, Writing – original draft, Visualization, Validation, Supervision, Software, Project administration, Methodology, Investigation, Funding acquisition, Formal analysis, Data curation, Conceptualization. **Md. Nasim Mahmud:** Writing – review & editing, Writing – original draft, Visualization, Validation, Software, Methodology, Investigation, Formal analysis, Data curation.

## Data available statement

Data were uploaded to the journal system as Supporting information for review and publication.

## Ethical statement

The ethical team of the Department of Marine Fisheries and Oceanography, Faculty of Fisheries, Aquaculture, and Marine Science, Sher-e-Bangla Agricultural University, Dhaka, Bangladesh highly recommended the research team of Roksana Jahan and Md. Nasim Mahmud to conduct the research entitled “Length-weight relationship, condition factors and reproductive biology of the spineless cuttlefish *Sepiella inermis* (Ferussac and d’Orbigny, 1848) in the southeastern regions of the Bay of Bengal, Bangladesh”. The authors have assured that the sampling of the species under investigation will be conducted in accordance with the international conventions on the use of animals in scientific research.

## Funding statement

The research was funded by 10.13039/501100006691Sher-e-Bangla Agricultural University Research System (10.13039/501100020392SAURES), 10.13039/501100006691Sher-e-Bangla Agricultural University, Bangladesh and University of Grand Commission (10.13039/100015747UGC), Bangladesh.

## Declaration of competing interest

The authors declare that they have no known competing financial interests or personal relationships that could have appeared to influence the work reported in this paper.
